# Measuring Treatment Outcomes in Women With Vulvodynia

**DOI:** 10.4021/jocmr526w

**Published:** 2011-04-04

**Authors:** Gary Ventolini

**Affiliations:** Department of Obstetrics and Gynecology, Wright State University Boonshoft School of Medicine, USA. Email: gary.ventolini@wright.edu

## Abstract

Vulvodynia or vulvar pain syndrome is a chronic, heterogeneous, and multifactorial gynecological condition with an estimated prevalence of 9 - 12%, broad and substantial effect on quality of life due to physical disabilities, psychological distress and sexual dysfunction. A rationale therapeutic approach for the treatment of vulvodynia is still under investigation. A review of treatment modalities proposed by most of the clinicians involved in managing these patients advocated initially utilizing non-invasive therapies and then to proceed gradually to more aggressive therapies. A multidisciplinary approach that includes behavioral science and neuroimaging is required and recommended. Additionally a team approach should be utilized to test and evaluate therapies including pelvic floor physiotheraphy, psychotherapy, microbiology and pharmacology. It is my hope that this review will assist in the understanding of vulvodynia and its measuring treatment outcomes and will provide a thrust in the right direction to once and for all clarify this complex multifactorial disorder affecting women.

**Keywords:**

Treatment; Vulvodynia; Women

## Introduction

Vulvodynia or vulvar pain syndrome is a chronic, heterogeneous, and multifactorial gynecological condition with an estimated prevalence of 9 - 12%, broad and substantial effect on quality of life due to physical disabilities, psychological distress and sexual dysfunction [1, 2]. Fifteen percent of women seen in the generalist practitioners’ office have reported pain on palpating areas of the vulvar vestibule. The condition may affect up to eighteen percent of the female population that includes Caucasians, African Americans, Africans and Hispanic women, particularly those who are sexually active. Women who present symptoms of vulvodynia vary in age between 16 to 80 years. The majority is aged between 20 and 50 [3]. The current and most widely accepted classification of vulvar pain as devised by the International Society for the Study of Vulvar Disease (ISSVD) recognizes vulvar pain related to a specific disorder (infections, inflammatory, neoplastic or neurologic) and vulvodynia (pain due to nonspecific etiology) [4].

## Therapeutic Modalities

A rationale therapeutic approach for the treatment of vulvodynia is still under investigation. A review of treatment modalities proposed by most of the clinicians involved in managing these patients advocated initially utilizing non-invasive therapies and then to proceed gradually to more aggressive therapies.

Initial medical approaches include the avoidance of all skin and mucosal irritants (like soap, perfumes, and deodorants), a diet containing low oxalates, scarce simple carbohydrates, and oral supplementation of calcium citrate. Additional measures are wearing cotton underwear, a trial of diverse relaxation techniques and psychological assessment with counseling and group support [5].

Topical treatments that have been reported as beneficial in multiple studies include topical gel anesthetics and estrogen cream particularly in perimenopausal women. Regional therapies including pudendal nerve block and pelvic floor muscle rehabilitation with or without biofeedback have also been recommended [6, 7].

Physical therapy has also shown to be effective in the treatment of vulvodynia [8]. It involves the evaluation of the patient pelvic musculature, joints, fascia and tendons. The function of other related organs such as bowel and bladder is evaluated as well. Most therapies employ a weekly one to two hour session focused on exercise for the pelvic floor and girdle, soft tissue mobilization, joint manipulation, muscle relaxation and general tone balance. However, standardization regarding the effective treatment approach is inexistent among therapists therefore outcomes cannot be accurately validated nor reproduced [9].

Because histological features of the vulvar epithelium can show an increased density of neural tissue and nonspecific inflammation, neuromodulators have been used. Oral medications include tricyclic antidepressants like amitriptiline, nortriptiline, and anticonvulsants like gabapentin. The above medications are usually initiated at a low dose and are increased over 2 - 4 week period. Opiates should be utilized only for short periods in acute settings and are not recommended as maintenance therapy [10].

Surgical excision of the vulvar tissue involved should be considered in unresponsive cases [11, 12]. Success from vestibulectomy varies between 65 and 90 percent but long-term relief is uncertain [13-15]. Generally, the vestibular epithelium is removed from the hymeneal ring to the posterior fourchette. This can be done unilaterally or bilaterally based on patients pain distribution. It is however still considered to be the most efficacious treatment option. Long-term follow-up studies are required to fully assess risks, side effects and implications of this procedure in the psychosexual well-being of the patient.

Laser ablation of the vulvar epithelium with the KTP-Nd: YAG Laser and the CO2 Laser, has been promoted as an option to the more invasive vestibulectomy. Since angiogenesis and increased nerve density are characteristics of vulvodynia, the laser is used to disrupt these histologic abnormalities and to promote collagen remodeling without altering the macroscopic anatomy. The KTP-Nd: YAG Laser and pulsed-dye Laser offer the advantage of being absorbed more readily by vasculature therefore they promote collagen remodeling. Results of Laser therapy for vulvodynia are comparable to vestibulectomy. Complete response has been reported in 62% and improvement in 92 % of the patients [14]. A recent report on KTP-Nd: YAG Laser use for vulvodynia with a 2 year follow-up found that 68% of the patients reported less pain with sexual intercourse and 29% reported no benefit [15].

Cognitive Behavior Therapy (CBT) has been applied to women with vulvodynia with encouraging results [16]. The objective of CBT is for the patient to learn to control their pain. Ter Kuile et al. found in a prospective, open-trial study that a reduction in dyspareunia was related to a general improvement in sexual functional level as well as increased control of the muscles of the pelvis [17]. Weijmar Schultz et al. have shown in a prospective, randomized study that CBT is comparable with surgery [18]. Bergeron and colleagues found in a small randomized, controlled study among 28 women who had been allocated for CBT, electromyographic biofeedback or vestibulecttomy, that surgery primarily led to a reduction in dyspareunia. Two years of follow-up results from this study showed that, even though surgery was better than CBT with regard to the intensity of pain on touching the vestibulum, the effect of CBT and surgery were comparable after two years in terms of self-reported pain during coitus [19]. Recently Desorchers et al. suggested based on their study that fear-avoidance variables and pain self-efficacy are significant predictors of topical and CBT treatment outcomes in women with provoked vestibulodynia (PV) [20].

Less well publicized methods of treatment such as acupuncture, local treatment using casaicin, ketoconazole, estrogen, steroids, niterfron, nifedipine and hypnosis are used to a lesser extent and with mixed results although without any major study reported. The clinician should be aware that a cure is not achieved in a short time even though the correct treatment is used. Improvement and reduction in pain may take several weeks. Therefore it is important to realistically inform and review the different options for treatment, to establish a multidisciplinary plan with a time frame and to assess results.

## Measuring Treatment Outcomes

The ISSVD definition of vulvodynia emphasizes three cardinal points: the localization of the pain, the involvement of the sensory nerves, and the multidimensional clinical picture. However, the emotional and psychological factors are not mentioned in the definition. Because of that it has often been omitted to study the connection between the physiological and the psychological factors that undoubtedly affect the condition.

Challenging features to appropriately measure treatment outcomes are: (1) the heterogeneous symptomatic manifestations, (2) the absence of a clear pathology, (3) the lack of an exclusively psychological explanation model, (4) the variable presence of clinically recognized objective signs (redness in the involved areas), and (5) the emotional superstructure that impacts vulvodynia.

## Instruments Used to Measure Outcomes

The Vulvar Vestibulitis Clinical Trial, Development of the National Institutes of Health, was a randomized, placebo controlled, double-blinded clinical trial to study the clinical efficacy of four medical treatments for vulvar vestibulitis syndrome (localized vulvodynia): topical lidocaine, oral desipramine, combined lidocaine and desipramine, and placebo cream and tablets [21]. The duration of study drugs lasted 12 weeks with postintervention follow-up at 16, 26 and 52 weeks. Clinical response was assessed by change in pain by numeric rating scale of a weekly Tampon Test compared with a number of measures with preexisting reliability/validity data or prior published experience in vulvodynia clinical trials, including change in overall daily pain intensity (24 hour numeric rating scale), the frequency of sexual intercourse (attempts per week), the change in intercourse pain numeric rating scale, vulvar algesiometer score, and the cotton swab test pain level by verbal reporting scale. In addition, during each study visit participants completed a battery of pain and health related quality-of-life measures recommended by IMMPACT including: the Brief Pain Inventory, Short Form McGill Pain Questionair (SF-MPQ), Profile of Mood States, and the Beck Depression Inventory. Therapeutic response of desipramine/lidocaine was estimated from preliminary reported data from the same group.

To be included in the trial, participants needed to fulfill Friedrichs Criteria for the diagnosis of vulvodynia, including tenderness localized within the vestibule confirmed by the cotton swab test modified from the technique of Bergeron et al. (Table 1).

**Table 1:** Friedrich Criteria for Vulvodynia Diagnosis

Score	Dyspareunia	Burning	Itching	Swab Test	Erythema
0	Absent	Absent	Absent	Negative	Absent
1	Mild Pain	Mild	Mild	Weakly Positive	Mild
2	Persistent	Moderate	Moderate	Positive	Moderate
3	With Intercourse	Severe	Severe	Strongly Positive	Severe

Adapted from Murina and coworkers dyspareunia, burning and itching are as described by the patient [22]. The swab test is performed with a cotton swab touched to the vestibule, and the degree of erythema is determinded by visual inspection of the vestibule by the examiner, the higher the score, the more severe the symptoms.

A pain diary card is usually filled out during week during the screening and baseline visit. Drug dose education phase treatment is initiated at a low daily dose for 2 weeks, increased to a medium for 2 weeks, and subsequently each dose for an additional week. The dosing schedule varies according to the medication used from once daily to up to three times a day. Drug maintenance phase and the target maintenance dose are tailored consequently. Dose tapering phase is usually necessary according to the pharmacokinetics of the medication used.

Pretreatment and posttreatment pain scores are measured on a discrete scale as follows: (1) the McGill Pain Rating Index, consisting of 15 pain descriptors ranging from 0 (none) to 3 (severe) describing pain for the previous month; (2) the McGill Visual Analog Scale of overall pain intensity for the past month, ranging from 0 (no pain) to 10 (worst possible pain); (3) the McGill Pain Intensity Scale modified to rate pelvic pain from 0 (no pain) to 5 (excruciating); (4) the Hamilton Depression Rating Scale (HAM-D), a standard interview-based measure of depression; and (5) the Hamilton Anxiety Rating Scale (HAM-A), a standard interview-based of anxiety [23].

The three-item subscale containing items such as “Rate the level of your pain at the present moment” on a scale of 0 (no pain) to 6 (very intense pain) was used.

Studies by gynecologists obtained the following ratings intended to correspond with Friedrichs three criteria for vulvar vestibulitis: the Speculum Rating, the Cotton-swab Rating, and the Erythema (redness of the skin) Rating.

The Speculum Rating is a four-point rating (i.e., absent (0), mild (1), moderate (2), and severe (3)), based on pain provoked at the introitus with speculum insertion. Narrow (2 cm width), prewarmed, metal speculums were used. Scores are calculated by averaging the two baseline rating. This measure has been shown to be valid and reliable [24].

The Erythema Rating is a four-point rating, using the same scale above, for the presence and degree of erythema.

The Cotton-swab Rating involves pain ratings of point tenderness provoked by cotton-swab palpation at each of six sites within the vulvar vestibule (i.e., clitoris, perineum, right and left labia minora, and right and left labia majora) and rated on a four-point scale: absent (0), mild (1), moderate (2), and severe (3). The measure has been shown to be valid and reliable [25].

The Pain Severity Subscale of the West Haven-Yale Multidimensional Pain Inventory (MPI) is a widely used multidimensional self-report instruments designed to assess chronic pain that has demonstrated reliability and validity [26].

The McGill Pain Questionnaire (MPQ) is a widely used measure of pain severity. The questionnaire comprises 20 sets of 3 - 5 descriptive words placed in order of increasing severity or intensity. The measure is used to assess three domains of pain: sensory, affective and evaluative. Subjects are instructed to circle one word within each set that best describes their pain. The measure has demonstrated acceptable reliability, and face, construct, discriminant and concurrent validity [27]. Total scores range from 0 to 78.

### Emotional function

The Beck Depression Inventory (BDI) is a psychometrically sound, widely used inventory of the cognitive, affective, motivational, and somatic symptoms of depression with adequate internal consistency, acceptable short-term test-retest reliability and good convergent validity with clinician ratings of depressive symptoms [28]. Higher scores reflect greater depressive symptom severity.

The Pain Anxiety Symptoms Scale (PASS) is a 40-item measure of fear of pain intended to measure four aspects of anxiety associated with clinical pain symptoms: cognitive anxiety, fear of pain, escape and avoidance, and physiological anxiety. The measure has been shown to be internally consistent and valid [29]. The frequency of each item (e.g., I think that if my pain gets too sever, it will never decrease) is rated on a six-point scale from 0 (never) to 5 (always). Higher scores reflect more frequent and more numerous anxiety responses.

### Treatment acceptability

Global treatment improvement is measured with the single item, “Up to what point do you feel your vulvar pain has improved following the treatment you received in this study?” Responses include: 0, worse; 1, no improvement; 2, little improvement; 3, moderate improvement; 4, great improvement; 5, complete cure.

Treatment satisfaction is measured with the single-item, “On a scale of 0 - 10, how would you rate your overall satisfaction with the treatment you received?” Responses ranged from “completely dissatisfied” to “completely satisfied”.

Treatment credibility is measured with a five-item scale adapted from Borkovec and Nau [30] and completed after treatment sessions one and ten (Le. Post-treatment). Items such as “How logical does the treatment seem to you?” were rated from 0 to 10.

### Sexual function

The Female Sexual Function Index (FSFI) is a 19-item multidimensional, self-report measure of sexual function. The measure has received empirical support for its reliability in several patient samples and for its ability to discriminate between samples of women with sexual dysfunction and healthy samples [31].

The FSFI includes both frequency items (“Over the past 4 weeks, how often did you experience discomfort or pain during vaginal penetration?”) and intensity items (“Over the past 4 weeks, how would you rate your level (degree) of discomfort or pain during or following vaginal penetration?”). Higher scores reflect better functioning.

## Bullet Points

- Despite many published studies on vulvodynia treatment during the past two decades, it remains difficult to recommend on specific treatment for any given patient.
- Based on recent prospective and randomized studies, attempts to recommend more evidence-based treatment are in place.
- In small non-randomized clinical trials, daily application of topical lidocaine and local corticosteroid-lidocaine injections provide considerable relief to women with provoked vestibulodynia.
- Multiple published studies have demonstrated the benefits of using pain medications like antidepressant, trycyclics and anticonvusants alone or combined.
- Vestibulectomy, evaluated in a randomized study, seems to be the most efficacious treatment option. Long-term follow-up studies are required to fully assess risks, side effects and implications of this procedure in the psychosexual well-being of the patient.
- A multidisciplinary approach that includes gynecology, microbiology, genetics, proteomics, biochemistry, pharmacology, behavioral science and neuroimaging is required and recommended to conclusively elucidate the pathophysiology of vulvodynia and to device the most appropriate therapy.

## Expert Opinion

My opinion, shared by a number of authors, includes the hypothesis that vulvodynia is a pathological alteration at the vaginal vestibule and the vulvovaginal milieu involving the mucosa, subcutaneous tissue, accessory sexual glands with their related circulatory and nervous system. This alteration is probably due to an insult, inflammation or infection and it is sustained by biological and chemical changes with consequent dysfunction of the vaginal vestibule, Bartholin, and Skene glands secreting function. These changes may be initiated by an insult or preceding infection or inflammation by bacteria, fungi, or virus that results in hypersensitivity leading to pelvic floor muscle dysfunction and recurrent pain. Vulvodynia may have an immune and genetic predisposition component leading to impaired neurological modulation of sensory C-afferent nociceptive processing and pathophysiologically distorted biochemical, immunological and behavioral responses to pain. There may be a potential role of psychosocial factors involved as it is the case like in other chronic pain conditions. Therefore a multidisciplinary approach that includes gynecology, microbiology, genetics, proteomics, biochemistry, pharmacology, behavioral science, and neuroimaging is required and recommended. Additionally a team approach should be utilized to test and evaluate therapies including pelvic floor physiotheraphy, psychotherapy, microbiology and pharmacology [19, 32-37].

This multidisciplinary approach will certainly allow us to frame a more specific and testable hypotheses regarding the etiology of this condition and to evaluate the most likely and significant factors leading to vulvodynia, furthermore it will certainly allow us to exclude other factors and to quality comorbid and psychiatric factors, triggers, and topical environment conditions favoring, promoting or perpetuating this condition. It is my hope that this review will assist in the understanding of vulvodynia and its measuring treatment outcomes and will provide a thrust in the right direction to once and for all clarify this complex multifactorial disorder affecting women.

### Multidisciplinary approach to evaluate patients with vulvodynia

1. The interaction of environment, psychosocial and co-morbid conditions implicated in vulvodynia.
2. The role that any insult or infections plays in the preceding of this condition.
3. The presence of any biochemical changes in the vulvovaginal milieu, and Bartholin and Skene glands in these patients.
4. The role of pelvic floor muscles and topical contributing factors.
5. The existence in these patients of any genetic predisposition that could impair the neurological modulation of sensory nociceptive processing.
6. The role of any biochemical, immunological or behavioral distortion in the response to pain.
7. Genetic testing to ascertain susceptibility genes involved including serological determination of behavioral makers.
8. Functional MRI (fMRI) to assess the functional and anatomical characteristics of subjects with vulvodynia compared to healthy women.
9. How current medications used in the treatment of vulvodynia target specific areas of the brain involved in pain responses.
10. The presence of any particular personality, behavioral feature or psychological factors involved in the propensity, instigation, promotion and perpetuation of chronic pain conditions like vulvodynia.
11. Evaluation of the patients response to pain (subjective, objective and the brain) prior to treatment and after pharmacological therapy used for vulvodynia.
